# Effect of Feeding Pomegranate (*Punica granatum*) Peel and Garlic (*Allium sativum*) on Antioxidant Status and Reproductive Efficiency of Female Rabbits

**DOI:** 10.3390/vetsci10030179

**Published:** 2023-02-22

**Authors:** Omnia Y. Abd-Elfadiel Hagag, Fawzy El-Essawy Younis, Rasha A. Al-Eisa, Eman Fayad, Nahla S. El-Shenawy

**Affiliations:** 1 Department of Zoology, Faculty of Science, Suez Canal University, Ismailia 41522, Egypt; 2 Physiology Department, Desert Research Centre, Cairo 11753, Egypt; 3 Department of Biology, College of Sciences, Taif University, P.O. Box 11099, Taif 21944, Saudi Arabia; 4 Department of Biotechnology, College of Science, Taif University, P.O. Box 11099, Taif 21944, Saudi Arabia

**Keywords:** pomegranate peel, garlic powder, rabbits, productive performance, biochemical parameters

## Abstract

**Simple Summary:**

Because rabbit meat has a good nutritional value, low levels of fat and cholesterol, and a high protein content, it may assist to alleviate meat shortages in developing countries. Since the 1970s, the processing of rabbit meat has developed into a highly specialized sector in various European nations, making Europe the world’s second-largest producer of rabbit meat, behind China. Livestock productivity is currently compromised by low animal reproduction efficiency. Adding pomegranate peel, garlic powder, or a combination of the two to the diet of does alter their weight, the number of offspring, reproductive performance, hematological indices, and many antioxidant indicators, as well as the liver and kidney functions. In conclusion, pomegranate is a promising substance to include in a rabbit’s diet, followed by garlic to boost reproductive efficiency.

**Abstract:**

Egypt’s animal protein shortfall cannot be overcome by expanding the production of large animals alone, but rather by increasing the production of highly reproducing animals in the livestock unit. The goal of this study was to examine how adding pomegranate peel (PP), garlic powder (GP), or a mixture of the two to the diet of does affect their weight, the number of offspring, reproductive performance, hematological indices, and several antioxidants indicators as well as the liver and kidney functions. A total of 20 adult and mature female mixed rabbits at age 4.5–5 months and averaging 3.05 ± 0.63 kg body weight, were allocated into four experimental groups (n = 5). The first group was provided with the basal diet and was considered as control animals, while the second, third, and fourth groups were fed the basal diet supplemented with PP 3.0%, GP 3.0%, and a mixture of PP 1.5% + GP 1.5%, respectively. After 2 weeks of feeding the experimental diets, natural mating with untreated bucks was carried out. The kits were weighed immediately after parturition, and then every week. The study found that rabbits fed with 3% PP led to a 28.5% increase in the number of kits at birth compared to the control group. As an effect of supplementing PP 3%, GP 3%, and PP 1.5% + GP 1.5%, the birth weight increased by 9.2%, 7.2%, and 10.6%, respectively, as compared to the control. Hemoglobin increased significantly in all treatment groups as compared to the control at the age of kit weaning. Lymph cells increased significantly in the rabbits that were fed with GP (3%) than in other groups and even the control. The results showed that creatinine levels were significantly decreased in the PP (3%) and GP (3%) than in control rabbits. The level of triglycerides significantly declines in groups treated with PP (3%) than in other treatment groups and the control. The addition of PP 3% or GP 3% increased the progesterone hormone. The addition of PP 1.5% + GP 1.5% improved the immunoglobulin IgG. The results of superoxide dismutase, catalase, glutathione, and total antioxidant capacity showed a significant decline in groups treated with GP (3%) than other treated groups. In conclusion, pomegranate is a promising substance to include in a rabbit’s diet, followed by garlic to boost reproductive efficiency.

## 1. Introduction

An additional 2 billion people should be fed over the next thirty years, necessitating a 70% increase in meat and milk production. Rising demand for livestock products in the future, driven by rising incomes, population growth, and urbanization, will place a huge strain on feeding capital [1]. Meat is derived from various traditional sources, such as poultry, beef, sheep, and goats, which, considering the high density of the livestock population, are rather inadequate to satisfy the increasing demand for animal protein. Protein deficiency has been identified as the key contributing factor to malnutrition [2].

Rabbit production in developing nations could help to reduce meat shortages, especially because rabbit meat has a high nutritional value, with minimal fat, cholesterol, and high protein content [3]. In some European countries, the processing of rabbit meat has increasingly become a highly specialized industry since the 1970s, making Europe the second largest producer of rabbit meat in the world after China [4]. Currently, low reproductive efficiency in animal production undermines livestock productivity. Several attempts to resolve this obstacle (poor reproductive performance) have contributed to the identification of oxidative stress (OS) as the culprit since OS directly or indirectly impairs the efficiency of animals [5].

Many attempts to examine the activity of natural antioxidants, particularly those of plant origin, have been made [1,6,7]. The availability of a significant number of by-products from the fruit and vegetable processing sector across the world encourages their use as a new source of feed in animal ration formulation [1]. Pomegranate by-products can be a reliable source of nutrients and antioxidants for livestock feeding, such as rabbits [8]. Several phenolic activities are already demonstrated in the pomegranate and its derivative portions [6]. Arils comprise 40% of the overall weight of the fruit, while the rest consists of seeds (10%) and peels (50%) [9]. The pomegranate peel (PP) has been associated with numerous health benefits. These beneficial by-products are functional elements in meats, nutraceuticals, and pharmaceuticals due to the high level of bioactive compounds in the peel and the documented health benefit [10].

The PP includes bioactive compounds that are abundant in the polyphenolic class of antioxidants, including tannins and flavonoids. In various pharmacological activities, such as anti-aging, anti-inflammatory, and anti-atherosclerotic activities, antioxidant activity has been suggested to play a vital function. Antioxidant supplementation’s ability to prevent free radical damage has made it a popular treatment strategy for lowering disease risk [11].

*Allium sativum* (garlic) is a popular spice in the Mediterranean region, as well as in rabbit diets and herbal medicine. As an antimicrobial, it plays a significant function in the prevention of a variety of diseases, extending from infections to heart disorders [12,13,14]. Garlic supplementation is crucial for broiler chicks because of its high immune-stimulating effects and beneficial effects on digestion in birds [15,16]. Despite the beneficial properties of *Punica granatum* L., there is currently limited research regarding its use in animal nutrition. In the current study, supplementation of rabbit diets with by-products (PP) or/and garlic powder (GP) was investigated for their effectiveness on female weight, the productivity of rabbits, hematological indices, of mother and kids rabbits, and several antioxidants indicators as well as the liver and kidney.

## 2. Materials and Methods

### 2.1. Chemicals

Total protein (TP), albumin (A), urea, and creatinine kits were provided from Diamond, 6th Street, Pyramids Garden-Giza, Egypt. Cholesterol (TC), HDL-c, and triglyceride (TG) kits were purchased from Spinreact, Ctra, Sta, Coloma 7, 17176 St, Esteve de Bas, Spain. Aspartate aminotransferase (AST) and alanine aminotransferase (ALT) kits as well as prolactin and progesterone ELISA (enzyme-linked immunosorbent assay kits were obtained from humans in the United States. However, Immunoglobulin G (IgG) and M (IgM) kits were taken from Bio-diagnostic, 29 El Tahrir, Ad Doqi, El Omraniya, Giza Governorate, Egypt.

### 2.2. Preparation of Diet

Pomegranate fruits (*P. granatum*) were collected from and included in the pomegranate farm in Ras Sudr Research, Egypt. Pomegranate peels were washed well in running water and dried in a drying oven at 80 °C for 3 days. Then, the dried peels were grounded using a grinder mill at Ras Sudr Research Station Laboratory. Garlic powder (GP) was collected from the Rajab Al Attar store in Cairo, Egypt.

### 2.3. Chemical Analysis of PP and GP

The samples of the PP and GP were subsequently analyzed in triplicate for crude protein (CP), ether extract (EE), moisture, and ash as described by the Association of Official Analytical Chemists [17] (Table 1). The following was written in the container pellet’s basic diet: rude fiber should be at least 22%, protein is 14%, fat is about 1%, and calcium is around 1%.

### 2.4. Experimental Design and Ethic

All experimental procedures were approved and performed in compliance with the guidelines of the Research Ethical Committee of the Faculty of Veterinary Medicine, Suez Canal University, Ismailia, Egypt (the approval No. 2019033). The present study was conducted at the experimental Ras Sudr Research Station, located in the South Sinai Peninsula in Egypt and belonging to the Desert Research Center, Ministry of Agriculture and Land Reclamation, during the period from November 2020 to January 2021. 

A total of 20 adult and mature female mixed of commercial rabbits as Northland and American (at age 4.5–5 months) averaged 3.05 ± 0.63 kg body weight, distributed into four experimental groups (n = 5) Each group has been housed in galvanized batteries (60 × 40 × 24 cm) provided with feeders and automatic drinkers. The occurrence of harmful infections was generally prevented by utilizing high-standard cleanliness and careful management, and rabbits were never treated with any type of systematic immunization.

The first group was provided with the basal diet and was considered as control, while the second, third, and fourth groups were fed the basal diet supplemented with pomegranate peel (PP) at 3.0%, garlic powder (GP) at 3.0%, or a mixture of PP 1.5% + GP1.5%, respectively (Table 2). The rabbits were kept in galvanized wire cages with a light/dark cycle of 12 h, a temperature of 24.5–27.5 °C, and relative humidity of 64–76%. The cages were given for manual feeders and nipple drinkers. They had free access to fresh water and feed ad libitum.

Natural mating with untreated bucks was conducted after two weeks after the beginning of feeding with the experimental diets. The buck/doe ratio was 1:5. Mating occurred between the hours of 8 a.m. and 9 a.m. Abdominal palpation was used to detect pregnancy 15 days after mating. After palpation, females that had not conceived were reintroduced to the same buck for another mating. The duration of experiment was 3 months (two weeks’ adaptation, 2 weeks to determine the abdominal palpation which was used to detect pregnancy after mating, a month of pregnancy, and a month for lactation till weaning.

### 2.5. Bodyweight

Each experimental rabbit in each group was weighed before mating and before parturition. The kits were weighed immediately after parturition, and then every week early in the morning before feeding and suckling to the nearest 10 g. The balance is Escali Digital Scale (The Winco SCAL-820 Scale with 8” Dial.) is perfect for weighing rabbits.

### 2.6. Blood Collection

For the does, blood samples were collected from the ear veins of each female rabbit after 21 days of the pregnancy, allowed to clot, and then centrifuged at 3000 rpm for 15 min to separate the serum. Serum samples were stored at −20 °C for further biochemical analysis. Frozen serum was thawed and assayed calorimetrically for all the biochemical parameters as described below. There was another patch collected in heparinized tubes for hematological estimation. The same two patches were collected from the kids after weaning.

### 2.7. Hematological Estimation

Hematological parameters fresh blood samples were collected in a heparinized tube from each female rabbit, weaning rabbit kits (4 weeks of age), and all parameters of the blood profile [erythrocyte cell counts (RBCs), white blood cells (WBCs), hematocrit (Hct), hemoglobin (Hb), mean corpuscular volume (MCV), mean corpuscular hemoglobin (MCH), mean corpuscular hemoglobin concentration (MCHC)], differential counts of white blood cells (WBCs), and platelet count (PLT) were measured in whole blood using the blood counter apparatus model Mindray Product BC-2800 Auto Hematology Analyzer (Guangdong Maikang Medical Co., Ltd, Guangdong, China).

### 2.8. Determination of Liver and Kidney Functions

According to Doumas et al. [18], the total protein (TP), albumin (A), globulin (G), and (A/G) ratio were assessed. USA kit (Elabscience; 14780 Memorial Drive, Suite 108, Houston, TX, 77079, USA) was used to determine the activity of both aspartate aminotransferase (AST) and alanine aminotransferase (ALT) according to Reitman and Frankel’s technique [19]. All the ELISA kits were obtained from Humans (Elabscience; 14780 Memorial Drive, Suite 108, Houston, TX, 77079, USA).

Spinreact (Ctra, Sta, Coloma 7, 17176 St, Esteve de Bas, Spain), the Spanish kit’s standard method, was used to measure serum total cholesterol (TC), high-density lipoprotein cholesterol (HDL-c), and triglyceride (TG) levels. Low-density lipoprotein cholesterol (LDL-c) was calculated using the Friedewald equation: LDL-c = TC-(HDL-c + TG/5) where (TG/5) = very-low-density lipoprotein cholesterol (VLDL-c). Kits were purchased from Spinreact, Spain.

The levels of urea and creatinine were determined using the methods outlined by Patton and Grouch [20] and Bartles et al. [21], respectively. Kits were taken from Biodiagnostic, 29 El Tahrir, Ad Doqi, El Omraniya, Giza Governorate, Egypt.

### 2.9. Estimation of Immunoglobulin and Hormones

Immunoglobulin G (IgG) and M (IgM) levels were determined using specific kits (Bio-diagnostic, Egypt). Prolactin and progesterone hormones were assayed by applying the ELISA method using ELISA kits ((Elabscience; 14780 Memorial Drive, Suite 108, Houston, TX, 77079, USA).

### 2.10. Oxidative Status and Antioxidants

Total antioxidant capacity (TAC) was determined using a Kit from (Cell Biolabs, 10225 Barnes Canyon Rd, San Diego, CA, USA). Superoxide dismutase (SOD) was determined using ELISA Kit and glutathione (GSH) was determined using ELISA Kit (Cusabio, 7505 Fannin St Ste 610-322 Houston, TX 77054, USA). Malondialdehyde and catalase (CAT) were determined using ELISA Kits (MyBioSource, Inc., San Diego, CA, USA).

### 2.11. Statistical Analysis

The data are represented as means of values ± S.E (n = 5). To compare the results, the One-way ANOVA test was applied to calculate basic statistic characteristics and to determine significant differences between the experimental and control groups. Differences were compared for statistical significance at the level of *p* ≤ 0.05 by using the statistical software SPSS.

## 3. Results

### 3.1. Bodyweight

**Figure 1:**
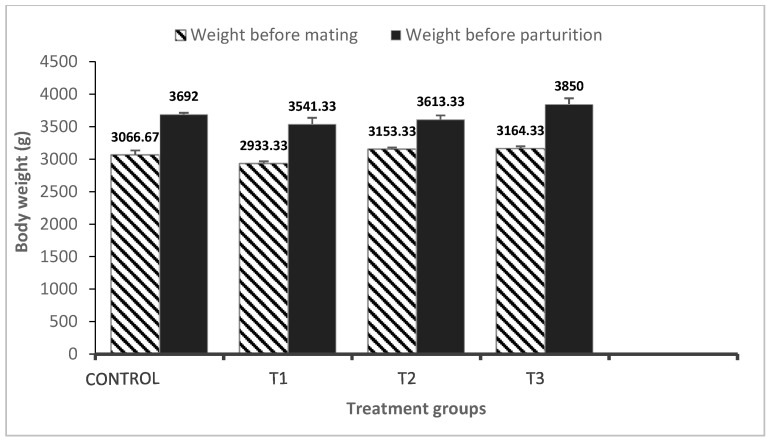
Effect of pomegranate peel and garlic powder separately or in combination on the weight of does. Values are presented as means ± SE (n = 5 litters). T1; pomegranate peel 3%, T2; garlic 3%, and T3; pomegranate peel 1.5% + garlic 1.5%. There are significant differences between before and after parturition.

Figure 1 depicts the effect of dietary PP, GP, and their combination on rabbit growth performance. The results showed that there was no significant difference in the weights of does before parturition between the treatments and the control group. The weight of rabbits in the combination group increased by 8% before parturition compared to the pomegranate group.

**Figure 2:**
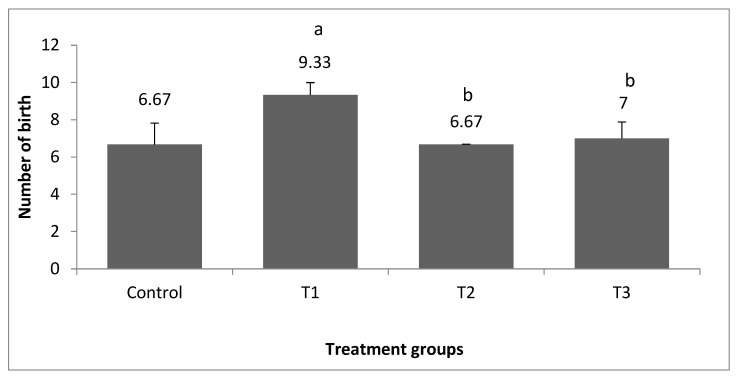
Effect of pomegranate peel and garlic powder separately or in combination on the number of kits at birth. Values are presented as means ± SE (n = 5 litters). T1; pomegranate peel 3%, T2; garlic 3%, and T3; pomegranate peel 1.5% + garlic 1.5%. ^a^ Significant difference as compared to the control group, and ^b^ significant difference as compared to T1.

**Figure 3:**
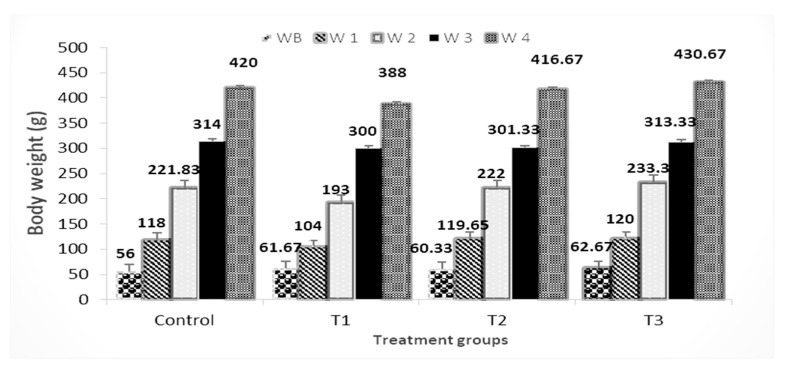
Effect of pomegranate peel and garlic powder separately or in combination on the weight of births at different times (W 1, W 2, W 3, and W 4 are presented weeks 1, 2, 3, and 4 respectively). Values are presented as means ± SE (n = 5 litters). T1; pomegranate peel 3%, T2; garlic 3%, and T3; pomegranate peel 1.5% + garlic 1.5%.

Figure 2 shows the number of kits at birth in each treatment and control group. When compared to those fed a control diet, the PP 3% group had a substantial (*p* < 0.05) increase in the number of kits at birth, almost 28.5%. When compared to other supplements, there was no significant difference in the weights of the rabbits born as a result of the rise in the number of rabbits born (Figure 3). When compared to the control, supplementing with PP 3%, GP 3%, and PP 1.5% + GP1.5 % increased birth weight by 9.2%, 7.2%, and 10.6%, respectively.

### 3.2. Hematological Parameters

Changes in hematological parameters after the birth of does are represented in Table 3. No significant differences were observed in any parameters of the T2 except for mean corpuscular hemoglobin (MCV) was increased significantly as compared to T1. T2 is significantly different from the T1 in the RBC counts. A significant decrease in MCV was recorded in the PP 1.5% + GP 1.5% group in comparison with the GP 3% group.

The number of WBCs shows a significant decrease in the GP group as compared to the control. On the other hand, a significant increase in WBCs was recorded in the PP 1.5% +GP 1.5% group as compared with other groups. Also, there was a significant increase in counts of mid and grand WBCs in the PP 1.5% GP 1.5% group compared to the other animals. In comparison with the control trend, highly significant (*p* < 0.01) increases in the content of platelets were detected in all experimental groups.

Table 4 shows the changes in hematological parameters after 4 weeks of weaning rabbit kits. HB content significantly increased (*p* < 0.05) in all treatment groups as compared to the control rabbits. The results showed the groups that were treated with PP 3%, GP 3%, and PP 1.5% + GP 1.5% increased HB by 9.9%, 10.86%, and 15.2% as compared to the control group, respectively. MCHC and WBCs decreased significantly in the PP 1.5% + GP 1.5% group as compared to the other groups and the control. However, a significant decrease in grand WBCs was noticed between all treatment groups and the control rabbits. However, lymph cells were increased significantly in the rabbits that were fed with GP 3% more than in other groups or even the control.

### 3.3. Liver and Kidney Functions

**Figure 4:**
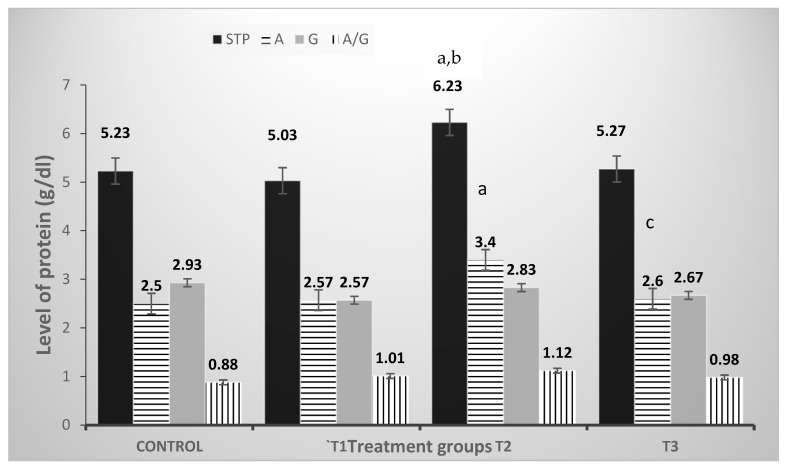
Effect of pomegranate peel and garlic powder separately or in combination on serum total protein (STP) of does. Data are presented as mean ± SE (n = 5 litters). T1; pomegranate peel 3%, T2; Garlic 3%, and T3; pomegranate peel 1.5% + garlic 1.5%. STP; serum total protein, A; albumin, G; globulin, A/G; albumin/globulin ratio. ^a^ Significant difference as compared to control, ^b^ significant differences as compared to T1, and ^c^ significant differences as compared to T2 (*p* < 0.05).

Data in Figure 4 demonstrated the effect of dietary PP, GP, and their combination supplementation on serum TP, A, G, and A: G ratio, as well as AST and ALT activity. No significant differences were noted in any of the serum protein parameters except for TP and A. A significant increase (*p* < 0.01) was observed in rabbits fed in the GP 3% group. The effect of GP 3% on TP was nearly 1.1-fold, while the effect of its addition on A was about 1.37-fold as opposed to all other groups.

**Figure 5:**
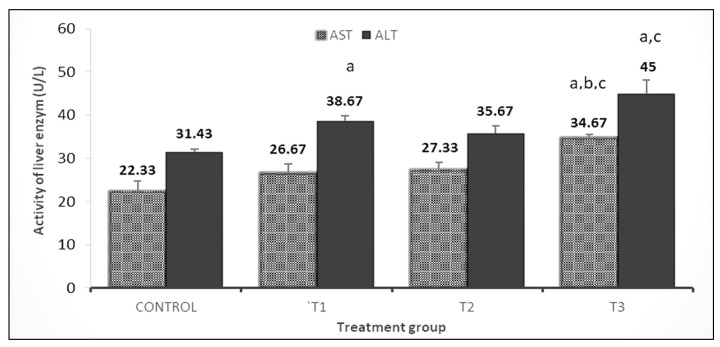
Effect of pomegranate peel and garlic powder separately or in combination on the activity of liver enzymes of does. Values are presented as means ± SE (n = 5 litters). T1; pomegranate peel 3%, T2; garlic 3%, and T3; pomegranate peel 1.5% + garlic 1.5%. AST; aspartate aminotransferase and ALT; alanine aminotransferase. ^a^ Significant difference as compared to control, ^b^ significant differences as compared to T1, and ^c^ significant differences as compared to T2 (*p* < 0.05).

Whereas results of liver function enzymes showed that AST activity significantly increased (*p* < 0.05) in the PP 1.5% + GP 1.5% group than in other groups. However, rabbits fed with PP 3% or PP 1.5% + GP 1.5% showed a significant increase in ALT activity by about 1.23-fold and 1.43-fold, respectively, as compared to the control group (Figure 5).

**Figure 6:**
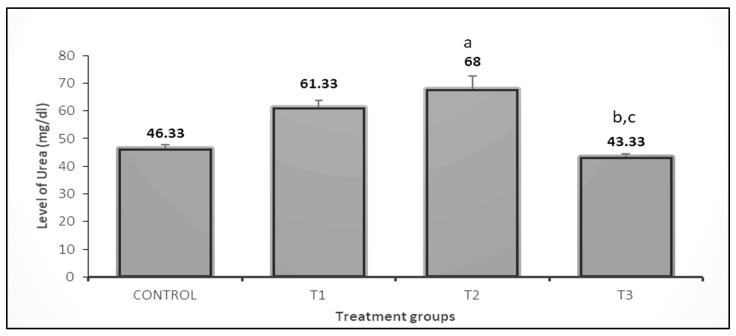
Effect of pomegranate peel and garlic powder separately or in combination on the level of serum urea of does. Values are presented as means ± SE (n = 5 litters). T1; T1; pomegranate peel 3%, T2; garlic 3%, and T3; pomegranate peel 1.5% + garlic 1.5%. ^a^ Significant difference as compared to control, ^b^ Significant differences as compared toT1, and ^c^ significant differences as compared to T2 (*p* < 0.05).

**Figure 7:**
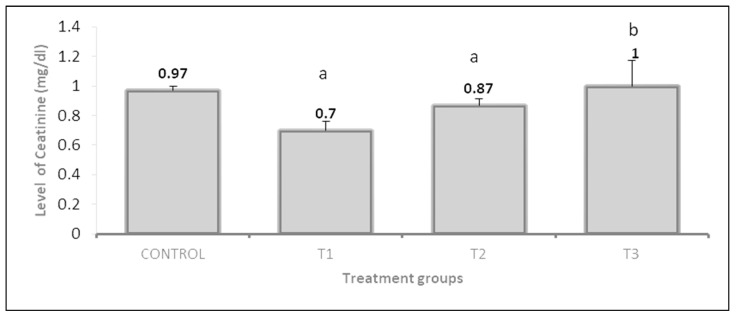
Effect of pomegranate peel and garlic powder separately or in combination on the level of serum creatinine of female rabbits. Values are presented as means ± SE (n = 5 litters). T1; pomegranate peel 3%, T2; garlic 3%, and T3; pomegranate peel 1.5% + garlic 1.5%. ^a^ Significant difference as compared to control, ^b^ Significant differences as compared to T1 (*p* < 0.05).

The effect of the PP 3% and GP 3% treatments on urea was about 1.32-fold, and 1.46-fold respectively, as compared to the control (Figure 6). On the other hand, the results showed creatinine levels significantly decreased in the PP 3% and GP3% than in control rabbits (Figure 7).

### 3.4. Effect of Supplementation on Lipid Profile

**Figure 8:**
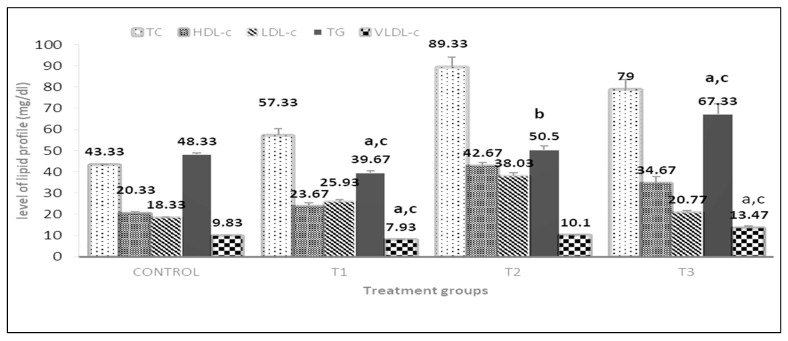
Effect of pomegranate peel and garlic powder separately or in combination on serum lipid profile of does. Values are presented as means ± SE (n = 5 litters). TC; The total cholesterol, TG; triglyceride, HDL-c; low-density lipoprotein, LDL-c; low-density lipoprotein, VLDL-c; very low-density lipoprotein. T1; pomegranate peel 3%, T2; garlic 3%, and T3; pomegranate peel 1.5% + garlic 1.5%. ^a^ Significant difference as compared to control, ^b^ Significant differences as compared to T1, and ^c^ significant differences as compared to T2 (*p* < 0.05).

The effect of dietary PP, GP, and their combination on the lipid profile is shown in Figure 8. The level of TC and HDL-c increased significantly (*p* < 0.05) in groups treated with GP 3% or the combination of both supplements as compared to the PP 3% group by about 1.56-fold, and1.33-fold, respectively, for TC and 1.77-fold, and 1.46-fold for HDL-c.

Whereas the level of TG and VLDL-c showed a significant (*p* < 0.01) decline in groups treated with PP 3% more than in other treatment groups and the control. The groups that are fed with GP3%, 1.5% + GP 1.5% increase the level of TG by about 1.27-fold and 1.69-fold, respectively, as compared to the PP 3% group. Moreover, the level of TG and VLDL-c showed a significant (*p* < 0.05) increase in groups treated with PP at 1.5% + GP 1.5% than in other treatment and the control groups.

### 3.5. Effect of Supplementation on Progesterone and Prolactin Hormones

**Figure 9:**
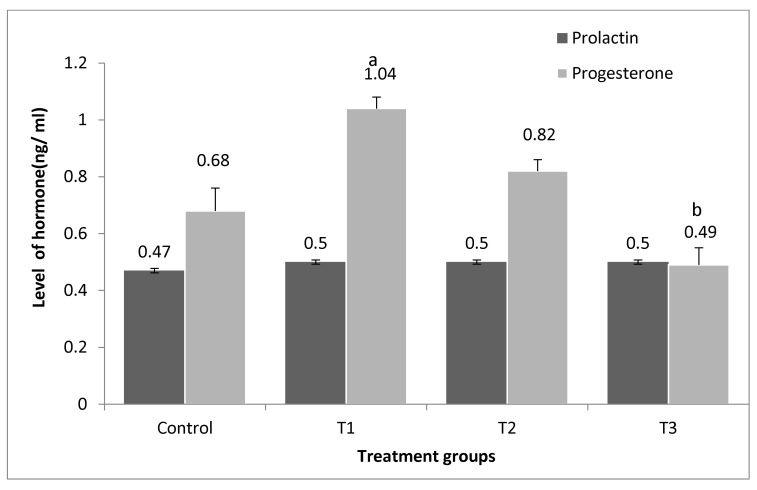
Effect of pomegranate peel and garlic powder separately or in combination on serum prolactin and progesterone levels of does. Values are presented as means ± SE (n = 5 litters). T1; pomegranate peel 3%, T2; garlic 3%, and T3; pomegranate peel 1.5% + garlic 1.5%. ^a^ Significant difference as compared to control, ^b^ Significant differences as compared to T1 (*p* < 0.05).

Prolactin hormone levels showed that no significant differences were found in the treatment and the control groups (Figure 9). As seen in Figure 9, showed a significant increase in the level of progesterone in the group treated with PP 3% by 52.9% and 34.6% more than 1.5% PP + 1.5% GP and the control, respectively.

### 3.6. Effect of Supplementation on Immunoglobulin IgG and IgM

**Figure 10:**
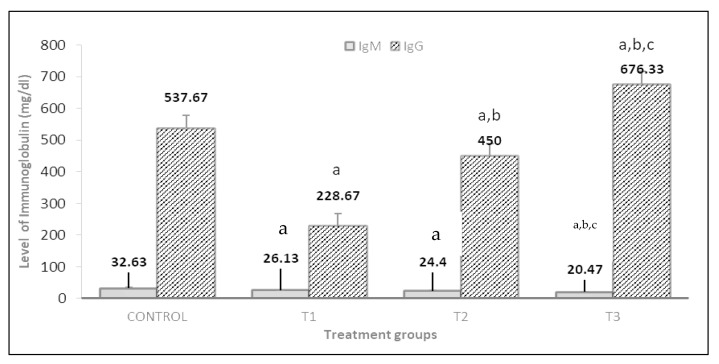
Effect of pomegranate peel and garlic powder separately or in combination on serum immunoglobulin IgG and IgM levels of does. Values are presented as means ± SE (n = 5 litters). T1; pomegranate peel 3%, T2; garlic 3% and T3; pomegranate peel 1.5% + garlic 1.5%. IgG is immunoglobulin G and IgM is immunoglobulin M. ^a^ Significant difference as compared to the control. ^b^ Significant differences as compared to T1 and ^c^ significant differences as compared to T2 (*p* < 0.05).

The level of IgM significantly decreased in the PP 1.5% + GP 1.5% treatment group than in other treatment groups as well as the control rabbits (Figure 10). In contrast, the PP 1.5% + GP 1.5% treatment group had significantly higher levels of IgG than the other treatment groups. The effect of PP 1.5% + GP 1.5% has increased the IgG by 66.19% and 33.46% as compared to the PP% and GP% groups, respectively.

### 3.7. Oxidative Stress/Antioxidant Status

The results of SOD, CAT, GSH, and TAC showed a significant decline in group treatments with 3% GP than other treatment groups and control (Table 5). In addition, the GSH and TAC levels showed a significant increase in the rabbits treated with the combination than in other treated groups. The results of MDA were found to be higher in the PP 3% group than in control rabbits (Table 5). However, the level of the MDA significantly increases in group treatment with GP by 3% than other treatment groups and control.

## 4. Discussion

The increased need for animal protein demands the utilization of the potential of minute livestock species and supports their inclusion in animal research and economic development programs, particularly in developing nations. Rabbit farming is classified among nonconventional breeding in Côte d’Ivoire [22]. This breeding is not commonly practiced, although it has very significant potential in terms of the productivity and nutritional value of the rabbit. One of the reasons for this state of affairs is its high production cost, linked largely to the high price of industrial feed for rabbits made from cereals and soybean meal [22]. The major purpose of this research would be how adding PP, GP, and their combinations affected does’ weight, number of offspring, and reproductive performance. Moreover, hematological parameters, serum immunoglobulins, liver and kidney functions, additionally, antioxidant effects were evaluated.

The effect of dietary PP and GP, as well as their combination, revealed that there was no significant difference in the weights of does before mating and before parturition between treatments. These results agree with those reported by Bahakaim et al. [23], who stated that adding PP powder to the rabbits’ diets during their pregnancy did not affect their final body weight or gain. Also, Bello et al. [24] found that when garlic was added to the diet, daily weight gain was not significantly different among rabbits of different sexes. These results contradicted an earlier study conducted on weaned rabbits by Onu and Aja [25], who reported that herbs mediated and restricted the growth and colonization of various pathogenic and non-pathogenic bacteria in the gut, resulting in an improved feed-to-meat ratio. This improvement by garlic supplementation may be due to providing some compounds that enhance digestion and absorption of some nutrients in the diets. Also, it may be attributed to the bioactive components (allicin) found in garlic that cause greater efficiency in the utilization of feed, resulting in enhanced growth [26].

Though there is a range of herbs that may be employed as natural growth boosters, the current findings indicated that the PP 3% group had a higher litter size, although mean birth weights were not significantly different throughout the trial when compared to the other groups. This increase in the number of kits at birth was about 28.51% as compared to the control. This result was partially consistent with the finding of Azoz and Basyony [27], who reported that when rabbits were fed a diet containing 1.5% PP, their litter size increased by around 40.86% when compared to rabbits fed a control diet. These could be attributed to antioxidants derived from natural sources, which are necessary for reproductive ability, immune response, and overall health [28]. Bahakaim et al. [23] found that adding PP powder or PP extract to the diet of a pregnant rabbit had no effect on the size of the litter at birth. There were no significant differences in litter size owing to the addition of PP on the 7th, 14th, or 21st days of breastfeeding. In this regard, Azoz and Basyony [27] reported that these changes could be traced back to the number of offspring per doe, but the decrease in pup weight is because young rabbits raised in bigger litters have less access to milk, resulting in reduced weight gain.

The dietary PP has no significant effects on any of the hematological parameters except the PLT count. One possible explanation [29] is that pomegranate juice drinking for a short period may boost erythropoiesis or prevent RBC degradation in healthy adults without generating large changes in metabolic health and inflammatory indices. El-Gindy [30] suggested that the WBC count was non-significantly reduced in pregnant rabbits fed with PP (1.5% and 3%), despite a non-remarkable increase in lymphocytes. The current data was in line with that of Riaz and Khan [31], who demonstrated that decrease in platelet aggregation and fibrinogen concentration, in a dose-dependent manner. The outcomes of hematological and coagulation assays raise the possibility that PP has an antianemic and cardioprotective effect. The garlic group had a significant increase in the RBC count in the current investigation. Onyimonyi et al. [32] reported that there was a significant elevation in RBC in the treatment groups of broilers with 1% and 5% GP in comparison to the control group. According to Fazlolahzadeh et al. [33], one explanation for the current study’s findings is that garlic contains various substances that may have a role in the function of organs involved in blood cell formation, such as the thymus, spleen, and bone marrow. According to Al-Jowari [34] and Onyimonyi [32], the effect of 1% GP on HB concentration, PCV, and PLT count in male rabbits has no significant differences. Manthou et al. [29] reported that the polyphenols in pomegranate juice may have protected HB from oxidative agents, resulting in enhanced HB levels in rabbit kids in the current study. This rise could be related to the activation of expression or catalytic activity of enzymes involved in GSH production that are known to be boosted by plant polyphenols [35]. The WBCs have the primary job of defending the body against foreign substances, which is accomplished by leukocytosis and antibody synthesis [36]. The current findings are consistent with those of El-Gindy [30], who discovered that PP therapy decreased the number of WBCs. When the quantity of PP in the diet was increased, the number of lymphocytes rose. Al-Jowari [34] found that the HB level, PCV, and PLT count in the 5% GP group were elevated as compared to the control rabbit. This could be due to a byproduct of garlic metabolism in the body that stimulates the kidney directly, causing erythropoietin synthesis and release. The present study is concerned with the effect of PP and GP on liver and kidney functions. The obtained results revealed the effect of dietary PP and GP in female rabbit diets on TP, A, G, and A: G ratio. Except for TP, there were no significant variations in any of these measures. A significant increase (*p* < 0.01) was observed in rabbits fed the GP 3% group as opposed to all other groups. These results agree with Nassrallah et al. [37], who reported that PP powder did not affect the albumin concentrations of rabbits.

These findings are consistent with those of the previous study [38], which reported that the A: G ratio didn’t vary significantly among the rabbit groups fed various levels of PP and the control. However, in contrast, Ibrahim et al. [38] noticed that supplementing with PP increased plasma TP, A, and G levels when compared to the control group. The current data corroborated with the previous study in which dietary allicin (10 mg/kg body weight) significantly improved protein metabolism by increasing TP and A levels [39]. As a result, several studies found that increasing TP concentration was linked to higher A concentration when garlic organosulphur compounds were present, which has a protective effect on liver function [40]. El-Katcha et al. [41] published similar findings on broiler chickens treated with allicin, as well as rabbits [26] treated with garlic. PP has also been demonstrated to be less prone to oxidation. This binding is linked to an increase in the resistance of LDL-c to oxidation [42]. The PP significantly increased blood HDL-c after 60 and 120 days of rabbit treatment, according to Abdel-Maksoud [43]. Perhaps this connection could help interpret the study’s results due to its brief length. According to Azoz and Basyony [27], all groups given varying amounts of PP (0.5, 1.0, and 1.5%) had significantly lower plasma triglycerides and VLDL-c than the control group. In the current study, the level of TC, LDL-c, HDL-c, TG, and VLDL-c increased significantly in groups treated with GP 3% as compared to the control.

However, Alagawany et al. [26] reported that there was no change in serum biochemical markers (TP, TC, TG, ALT, and AST) between broilers fed a garlic-enriched diet and those fed a control diet. Hypertriglyceridemia may be caused by a decrease in lipoprotein lipase activity combined with an increase in hormone-sensitive lipase activity, resulting in decreased TG uptake from the circulation [44]. Codoñer-Franch et al. [45] reported that anti-oxidant enzymes are destroyed by excess synthesis of oxidized low-density lipoproteins (ox-LDL), which inhibits SOD expression, and could also be responsible for decreased SOD activity. Fed a base diet with varying doses (ranging from 1,000 to 1,500 mg/kg) of whole-pomegranate extract for 60 days in the summer to the rabbits. LPO, AST, ALT, TC, TG, and WBC, which were negatively impacted by summer heat stress in the control rabbits, were dramatically reduced by the extract [46]. The presence of many phenolic components (ellagic acid, punicalin, and punicalagin) rather than a single pure polyphenol accounts for the pomegranate’s superior antioxidant action [47]. Pomegranate was a good source of natural antioxidants due to its exceptional effectiveness in scavenging hydroxyl and superoxide anion radicals. Garlic, as previously reported, includes a diverse range of phytochemicals. These phytochemicals are detoxified or metabolized in the liver after ingestion. The increased activity of the liver as a result of the increased garlic levels accounts for the observed rise in these blood chemistry indices of the liver [32].

Biomarkers for kidney function include urea and creatinine. The results showed that urea levels were significantly increased in the PP and GP groups compared with the control. On the other hand, creatinine levels significantly decreased in the PP 3% and GP 3% than control. This data is in agreement with that of Ibrahim et al. [38], who found that when rabbits were fed different amounts of PP, their creatinine levels decreased significantly. This result agrees in part with Abdel-Wareth et al. [48] findings. When compared to non-garlic-added groups, dietary supplementation with garlic oil reduced serum urea, creatinine, and urea-nitrogen activity.

Progesterone hormone levels were significantly increased in the PP group than in the control and the GP showed an increase in progesterone hormone. There were no significant variations in prolactin hormone levels between the treatment and the control groups. As reported by DeMayo et al. [49], progesterone and oestradiol are key hormones for sexual maturity and reproduction. The results follow the results of Liebler [50] who used vitamin E, where the protective effect of antioxidants against LPO in the cell membrane could explain the improved reproductive function in PP treatment.

The results of the GP group are also similar to El-Ratel et al. [39], who were treated with allicin at both levels (5 and 10 mg per kg). In comparison to the control, there was an increase in blood progesterone levels at mating, mid-pregnancy, and 7-days post-partum. Also, both allicin levels 7 days after birth did not affect a significant increase in prolactin concentration. However, garlic extract was found to enhance gonadotropin secretion and ovarian hormones by activating the anterior pituitary [51].

The current study also examined the effects of PP and GP on rabbit immunity. The IgM levels in the PP and GP groups did not change. Meanwhile, IgG significantly declined in the PP group than in the control. The obtained results are supported by El-Sissi et al. [52]. At 12 weeks of age, rabbits fed high concentrations of PP and PP extract showed a significant decrease in IgG. Gracious et al. [53] reported that feeding pomegranate fruit rind powder orally increased typhoid antigen-antibody levels. Li et al. [54] demonstrated that protein and amino acid deficiencies have been known to disturb immune function, as amino acids such as arginine, glutamine, and cysteine. They play a crucial part in the immune system’s reaction by activating lymphocytes and macrophages, controlling gene expression, and synthesizing specific proteins such as cytokines, antibodies, and cytotoxic substances. Relatively consistent with Alagawany et al. [26], they found that garlic in rabbit diets increased IgG concentrations linearly and quadratically, but IgM concentrations were unaffected by GP in contrast to the control group.

The antioxidant parameters (CAT, SOD, GSH, and TAC) showed a significant decline as compared to the control. MDA results, on the other hand, were found to be higher in the PP and GP groups than in the control rabbits. Hermes-Lima et al. [55] postulated that activation of antioxidant defenses, in which real oxyradical generation should decrease, is a protective mechanism against OS generated by physiological stress circumstances. Excess H_2_O_2_ in the intermembrane gap may pass through the external mitochondrial membrane and alter the redox status of the entire liver by lowering the GSH level, according to El-Hafidi et al. [56]. The PP can chelate metal ions like Cu^2+^ and Fe^2+^ that catalyze free radical production reactions. It also meets the structural requirements for optimal antioxidant and/or scavenging activity [57]. Reduced SOD activity, a sign of OS, was also a factor in the higher MDA levels observed in this study, according to Nabil et al. [58]. Furthermore, El-Hafidi et al. [56] found that decreased CAT activity in the sucrose-fed rats SFR hepatic homogenate may contribute to higher levels of LPO and protein carbonylation in whole liver cells. After the first month of pregnancy, Erisir et al. [59] investigated the levels of oxidants and antioxidants in sheep, finding lower CAT activities and enhanced GSH levels. It can also be considered that the elevation of urea in PP3% and GP3% groups affects antioxidant status, as Ahmed and Ali [60] explain that increased serum urea and creatinine accelerate renal LPO with the reduction in renal GSH content, CAT, and glutathione reductase.

According to our knowledge, there is no data available on the combination of GP and PP as supplemented diet for the rabbit. Therefore, it was difficult to compare the present study with previous investigation. Each treatment has certain effect on the different biochemical parameters.

In conclusion, one of the most promising additives in the rabbit diet is PP, as it improved the fertility of does and increasing the number of kits, resulting from the elevation of the progesterone hormone. It also reduced triglycerides. Pomegranate has the capacity to combat hyperlipidemia with therapeutic benefits. As for the garlic supplement, it had good results in increasing fertility. Also, it affected increasing the total protein, albumin, and lipid profile. As shown, the combination of pomegranate peel and garlic negatively affected the progesterone hormone and the lipid profile, they had a positive effect on immunoglobulin. All the supplements had an improvement in the blood profile of the offspring, especially hemoglobin.

## Figures and Tables

**Table 1:** Chemical analysis of pomegranate peel and garlic powder.

Chemical Analysis (%)	PP	GP
CP	17.51 ± 0.1	18.6 ± 0.2
Moisture	13.80 ± 0.3	9.44 ± 0.2 ^a^
Ash	4.40 ± 0.1	3.7 ± 0.1
EE	3.65 ± 0.1	2.52 ± 0.1

Data presented as mean ± SE (n = 5). CP; crude protein, and EE; ether extract. ^a^ Significant difference as compared to PP.

**Table 2:** Ingredients of the experimental diets.

Ingredients %	Basal Diet (BD)	Pomegranate (PP-3%)	Garlic Powder (GP-3%)	Both Materials (PP1.5% + GP-1.5%)
Yellow corn	5	5	5	5
Wheat bran	22	22	22	22
Barley	12	12	12	12
Clover hay	30	28.5	28.5	28.5
Hay	4	2.5	2.5	2.5
Soybean meal (20%CP)	23	23	23	23
Pomegranate peel	0	3	0	1.5
Garlic powder (GP)	0	0	3	1.5
Limestone	1.15	1.15	1.15	1.15
Di-calcium phosphate	0.5	0.5	0.5	0.5
DI-Methionine	0.2	0.2	0.2	0.2
Anti-aflatoxin + anticoccidial1	0.5	0.5	0.5	0.5
Vitamin and minerals premix *	0.3	0.3	0.3	0.3
Di-sodium	0.5	0.5	0.5	0.5
NaCl	0.35	0.35	0.35	0.35

* Vitamin–mineral premix: Each 2.5 kg contains vit. A (10,000,000 IU), vit. D3 (2,000,000 IU), vit. E (10 g), vit. k3 (1000 mg), vit. B1 (1000 mg), vit. B2 (5 g), vit. B6 (1.5 g), pantothenic acid (10 g), vit. B12 (10 mg), niacin (30 g), folic acid (1000 mg), biotin (50 g), Fe (30 g), Mn (60 g), Cu (4 g), I (300 mg), Co (100 mg), Zn (50 g).

**Table 3:** Effect of pomegranate peel and garlic powder separately or in combination on blood profile of does.

Groups	Parameters of Blood Profile													
	RBC (10^6^/μL)	HB (g/dl)	HCT (%)	MCV (FL)	MCH (Pgs.)	MCHC (g/dl)	RDW-CV %	RDW-SD (FL)	WBC (10^3^/μL)	Lymph (10^3^/μL)	Mid (10^3^/μL)	Grand (10^3^/μL)	PLT (10^3^/UL)	MPV (FL)
Control	5.61 ± 0.37	9.37 ± 0.58	32.13 ± 2.34	57.23 ± 0.58	16.37 ± 0.25	29.17 ± 0.48	14.9 ± 0.10	29.47 ± 0.33	13.9 ± 1.09	3.67 ± 0.45	0.63 ± 0.07	9.0 ± 1.82	322 ± 8.69	8.13 ± 0.32
T1	4.83 ± 0.21	8.70 ± 0.90	25.27 ± 0.03 ^a^	58.03 ± 0.64	16.30 ± 1.7	29 ± 0.96	14.7 ± 0.60	29.53 ± 1.27	11.27 ± 1.12	3.23 ± 1.16	0.70 ± 0.12	6.6 ± 0.41	236 ± 18.61 ^a^	8.23 ± 0.27
T2	6.39 ± 0.71 ^b^	9.63 ± 0.41	37.30 ± 4.63	62.33 ± 2.28 ^b^	16.80 ± 0.72	26.17 ± 1.99	14.0 ± 0.83	31.07 ± 1.39	8.57 ± 0.43 ^a^	3.97 ± 0.03	0.40 ± 0.06	4.2 ± 0.38 ^a,b^	288.7 ± 14.19	8.10 ± 0.30
T3	5.79 ± 0.29	9.80 ± 0.12	31.53 ± 1.21^b^	54.63 ± 1.25 ^c^	16.67 ± 0.13	29.97 ± 0.27	14.6 ± 0.41	27.33 ± 0.67 ^a^	16.73 ± 1.05 ^b,c^	4.87 ± 0.32^c^	1.30 ± 0.06 ^a–c^	11.0 ± 0.58 ^b,c^	224.3 ± 7.06 ^a,c^	7.67 ± 0.15

Values are presented as means ± SE (n = 5 litters). T1; pomegranate peel 3%, T2; garlic 3%, and T3; pomegranate peel 1.5% + garlic 1.5%. ^a^ Significant difference as compared to control, ^b^ significant differences as compared to T1, and ^c^ significant differences as compared to T2 (*p* < 0.05). RBC, red blood cells; HB, hemoglobin; HCT, hematocrit, MCV, mean corpuscles volume; MCH, mean corpuscle hemoglobin, MCHC, mean corpuscular hemoglobin concentration; RDW-CV, red cell distribution width; RDW-SD, red cell distribution width standard deviation; WBC, weight blood cells; lymph, lymphocytes; Mid, cells include less frequently occurring and rare cells correlating to monocytes, eosinophils, basophils, blasts and other precursor white cells that fall in a particular size range, Grand, granulocytes (Absolute Neutrophil Count); PLT, platelets; and MPV, mean platelet volume.

**Table 4:** Effect of pomegranate peel and garlic powder separately or in combination on blood profile of rabbit’s kits after 4 weeks.

Groups	Parameters of Blood Profile													
	RBC (10^6^/μL)	HB (g/dl)	HCT (%)	MCV (FL)	MCH (Pgs.)	MCHC (g/dl)	RDW-CV %	RDW-SD (FL)	WBC (10^3^/μL)	Lymph (10^3^/μL)	Mid (10^3^/μL)	Grand (10^3^/μL)	PLT (10^3^/UL)	MPV (FL)
Control	5.11 ± 0.16	8.2 ± 0.15	30.4 ± 0.87	62.5 ± 2.16	17.3 ± 0.85	27.8 ± 0.6	17 ± 1.3	33.9 ± 1.68	7.7 ± 0.32	1.83 ± 0.32	0.4 ± 0.03	5.5 ± 0.35	287 ± 32.13	8.3 ± 0.09
T1	5.7 ± 0.56	9.1 ± 0.45	35.6 ± 4.23	61.6 ± 1.3	16.8 ± 0.2	27.53 ± 0.28	16.6 ± 0.28	34.5 ± 1.4	5.6 ± 0.6a	2.4 ± 0.12	0.37 ± 0.09	2.7 ± 0.38 ^a^	348 ± 77.7	8.3 ± 0.27
T2	5.36 ± 0.16	9.20 ± 0.0.6 ^a^	32.97 ± 0.39	60.3 ± 1.03	17.1 ± 0.55	27.9 ± 0.47	17.5 ± 0.48	38.6 ± 0.85	7.6 ± 1.15	4.0 ± 0.49 ^b^	0.43 ± 0.09 ^a^	3.1 ± 0.58 ^a^	430.3 ± 72.80	7.9 ± 0.26
T3	5.55 ± 0.12	9.67 ± 0.50 ^a^	33.43 ± 1.66	58 ± 1.81	17.1 ± 0.6	29.1 ± 0.45b	20.6 ± 0.84 ^bc^	37.9 ± 0.81	3.9 ± 0.21 ^ac^	1.8 ± 0.03 ^cb^	0.17 ± 0.03 ^ac^	1.9 ± 0.17 ^a^	334.6 ± 60.3	8.1 ± 0.20

Values are presented as means ± SE (n = 5 litters). T1; pomegranate peel 3%, T2; garlic 3%, and T3; pomegranate peel 1.5% + garlic 1.5%. ^a^ Significant difference as compared to control, ^b^ significant differences as compared to T1, and ^c^ significant differences as compared to T2 (*p* < 0.05). RBC, red blood cells; HB, hemoglobin; HCT, hematocrit, MCV, mean corpuscles volume; MCH, mean corpuscle hemoglobin, MCHC, mean corpuscular hemoglobin concentration; RDW-CV, red cell distribution width; RDW-SD, red cell distribution width standard deviation; WBC, weight blood cells; lymph, lymphocytes; Mid, cells include less frequently occurring and rare cells correlating to monocytes, eosinophils, basophils, blasts and other precursor white cells that fall in a particular size range, Grand, granulocytes (Absolute Neutrophil Count); PLT, platelets; and MPV, mean platelet volume.

**Table 5:** Effect of pomegranate peel and garlic powder separately or in combination on serum Some antioxidant parameters levels of does.

Groups	MDA (nmol/mL)	SOD (U/mL)	CAT (ng/mL)	GSH (ng/mL)	TAC (ng/mL)
Control	0.50 ± 0.057	248.00 ± 15.51	16.84 ± 1.77	195.50 ± 6.12	17.23 ± 0.89
T1	3.51 ± 0.61 ^a^	155.50 ± 6.12 ^a^	3.42 ± 0.84 ^a^	126.00 ± 7.35 ^a^	6.49 ± 0.82 ^a^
T2	10.82 ± 1.98 ^a^	54.50 ± 10.21 ^a,b^	0.63 ± 0.12 ^a^	28.00 ± 2.45 ^a,b^	0.86 ± 0.04 ^a,b^
T3	0.93 ± 0.05 ^a–c^	221.50 ± 8.57 ^b,c^	9.10 ± 029 ^a–c^	157.00 ± 6.53 ^a–c^	12.00 ± 1.63 ^a–c^

Values are presented as means ± SE (n = 5 litters). T1; pomegranate peel 3%, T2; garlic 3%, and T3; pomegranate peel 1.5% + garlic1.5%. Lipid peroxidation (MDA), superoxide dismutase (SOD), catalase (CAT), glutathione (GSH), and total antioxidant capacity (TAC). ^a^ Significant difference as compared to control, ^b^ significant differences as compared to T1, and ^c^ significant differences as compared to T2 (*p* < 0.05).

## Data Availability

Not applicable.
